# A systematic review on the culture methods and applications of 3D tumoroids for cancer research and personalized medicine

**DOI:** 10.1007/s13402-024-00960-8

**Published:** 2024-05-28

**Authors:** Jessica Kalla, Janette Pfneissl, Theresia Mair, Loan Tran, Gerda Egger

**Affiliations:** 1 Department of Pathology, Medical University of Vienna, Vienna, Austria; 2 Ludwig Boltzmann Institute Applied Diagnostics, Vienna, Austria; 3 Comprehensive Cancer Center, Medical University of Vienna, Vienna, Austria

**Keywords:** Cancer, 3D models, Preclinical models, Organoids, Tumoroids, Precision medicine, Co-culture, Bioprinting, Fluidic devices

## Abstract

Cancer is a highly heterogeneous disease, and thus treatment responses vary greatly between patients. To improve therapy efficacy and outcome for cancer patients, more representative and patient-specific preclinical models are needed. Organoids and tumoroids are 3D cell culture models that typically retain the genetic and epigenetic characteristics, as well as the morphology, of their tissue of origin. Thus, they can be used to understand the underlying mechanisms of cancer initiation, progression, and metastasis in a more physiological setting. Additionally, co-culture methods of tumoroids and cancer-associated cells can help to understand the interplay between a tumor and its tumor microenvironment. In recent years, tumoroids have already helped to refine treatments and to identify new targets for cancer therapy. Advanced culturing systems such as chip-based fluidic devices and bioprinting methods in combination with tumoroids have been used for high-throughput applications for personalized medicine. Even though organoid and tumoroid models are complex in vitro systems, validation of results in vivo is still the common practice. Here, we describe how both animal- and human-derived tumoroids have helped to identify novel vulnerabilities for cancer treatment in recent years, and how they are currently used for precision medicine.

## Background

### Model systems for research

One of the biggest challenges in science is the representative modeling of multicellular tissues, dynamic interactions, and the complex mechanisms underlying disease. Therefore, scientists mostly rely on different model systems ranging from cell lines to animal models, to representative models derived from primary tissue [1].

2D cell lines have been established from a diverse range of malignant diseases but also from a selection of cell types in the healthy human body and are a widely used model system. Because of easy handling for in vitro cell culture and simple introduction of genetic modifications, they can deliver meaningful insights on specific research questions [2]. However, these cell lines consist of only one cell type optimized for long-term culture. Additionally, as the cultivation in a monolayer can alter the shape, polarization, and global signalling of cells, 2D cell lines do not adequately reflect the physiological situation in organs [3, 4]. Therefore, research relying solely on cell lines does not successfully translate from bench to bedside [5, 6].

Animal models, which better reflect the cellular complexity and molecular crosstalk of organs in the human body, are frequently used to study cells in their physiological context. However, animals and humans are still inherently different so that not all biological processes can be modeled accurately. Moreover, animal models are more complicated and time consuming to work with than in vitro cell lines, in addition to the ethical issues connected to the use of animals for research [7].

### Organoids

Organoids are one model system that combines several advantages of in vitro 2D cell culture and in vivo animal models [8]. Even though commonly used 3D spheroids are a promising tool for cancer research, they are cell line-derived aggregates that do not self-organize or differentiate into multiple cell types [9]. In contrast, organoids are 3D self-organizing cell clusters that can be derived from embryonic stem cells, tissue-specific adult stem cells, or from induced pluripotent stem cells (iPSCs) from both animals and humans. These stem cells, which are commonly embedded in an extracellular matrix (ECM), can then further differentiate into different types of epithelial cells specific for the tissue of interest [10–14]. In addition to stem cell-derived organoids, tumor-derived organoids, so called tumoroids, have been successfully generated for various cancer entities and provide valuable tools for cancer research.

#### Establishment and culture

Although a lot of research has been done on 3D models derived from stem cells in the past [15], the group of Hans Clevers pioneered the successful establishment of organoids in the modern era [14]. These organoids were generated in 2009 from murine intestinal stem cells, and since then many different research groups have adapted the establishment protocol to generate an extensive collection of organoid models from both animals and humans [16].

Typically, primary surgical resection material is cut into small fragments using scalpels, followed by an enzymatic digestion to dissociate the tissue into single cells or small cell clumps [17, 18]. However, as access to tissue samples can be anatomically and logistically limited, stem cells derived from fine needle aspirates of tissues [19–21], fluid samples such as urine [22], ascites [23], broncho-alveolar lavage fluid [24], and bile [25], or enriched circulating tumor cells (CTCs) [26] might provide a minimally invasive alternative for organoid generation.

Organoids are mostly cultivated in 3D ECM hydrogel domes such as basement membrane extract, Matrigel® [27], or Geltrex®, in addition to a variety of xeno-free synthetic hydrogels [28]. Organoid culture medium provides a variety of niche-specific growth factors matched to the tissue of origin to ensure optimal growth conditions [17, 29, 30]. Common key components include activators of the canonical WNT pathway (R-Spondin), MAPK pathway (EGF), and inhibitors of TGF-β (A83-01) as well as BMP signaling (Noggin) to allow stem cell renewal and proliferation [11, 14, 17].

Stable organoid lines can be expanded long-term (> 10 passages) in vitro, and cryopreserved [31]. Of note, even though conventional submerged organoid cultures exclusively enrich for epithelial cells and fail to retain stromal components [32], organoids do recapitulate the tissue architecture on a microscale, and can mimic the function of the organ they resemble, while stably maintaining mutational and epigenetic states in line with cellular signaling [33–35].

#### Organoids for cancer research: tumoroids

**Fig. 1:**
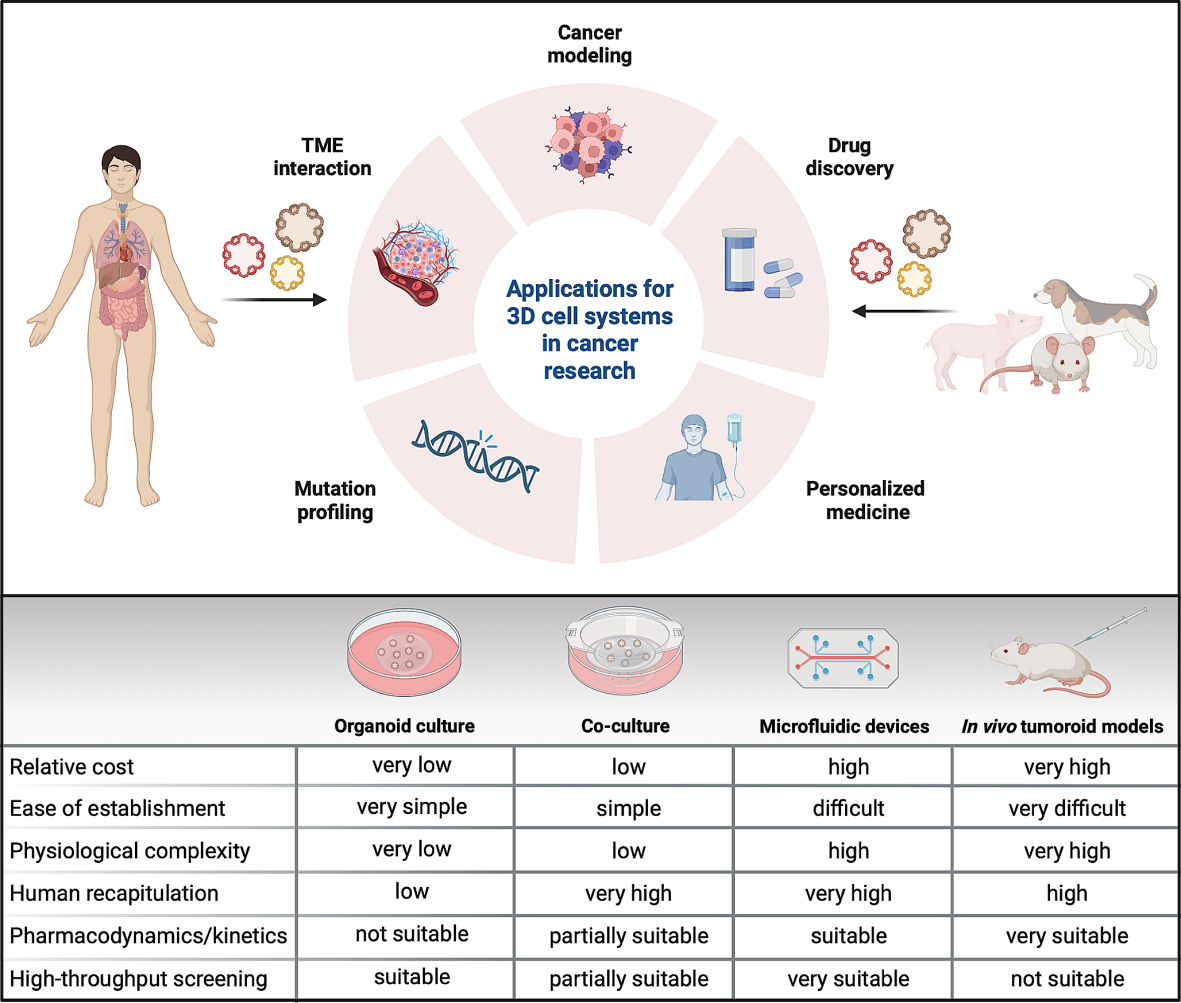
3D model systems in cancer research. Applications of both human- and animal-derived tumoroids in cancer research (top). Tumoroids can be used for either cancer modeling with a focus on the tumor microenvironment (TME), or as a tool for personalized medicine and drug discovery based on the mutational landscape of patients. Comparison of different 3D model systems, including organoids, co-culture models, microfluidic devices, and in vivo models, highlighting the advantages and disadvantages of each model (bottom). Created with BioRender.com

Organoids are used in many different research areas, including reconstructive medicine, evolutionary biology, and pathogen-host interactions, summarized in previously published reviews [36–41]. In recent years, tumoroids derived from both animals and humans have been intensively used for preclinical cancer modeling, with a focus on cancers originating from epithelial cells (Fig. 1). However, efforts have been made to also establish tumoroids from non-epithelial cancers and rare cancer types, for example melanoma [42], glioblastoma [43, 44], rhabdomyosarcoma [45], epithelioid sarcoma [46], and rhabdoid cancer [47].

Disease modeling using tumoroids allows for the investigation of different hurdles of oncology such as tumor initiation and driver events of cancer, tumor heterogeneity or development of efficient therapies [10, 48–50]. By establishing healthy organoids and altering cancer type specific genes in vitro, the transformation from healthy to cancerous cells can be studied in a 3D model focusing on different tissues and diseases. Additionally, by generating organoids and tumoroids directly from both healthy and tumor tissue, cancer driver mutations can be identified and used to discover patient-specific therapeutic targets [33, 51]. This way, organoids and tumoroids could be used for both answering basic questions of tumorigenesis and developing a personalized medicine approach to help advance the field of cancer research.

Given recent advancements in organoid technologies, we here summarize the latest developments in the field, outlining various methods and practical applications of these innovative model systems. We will focus on current uses for cancer research including both human- and animal-based methods. Furthermore, we will highlight recent work that has improved preclinical cancer models by using tumoroids in combination with other cell types to model the tumor microenvironment (TME). Moreover, studies focusing on advanced 3D culture systems based on fluidic devices and bioprinting, and grafting tumoroid models into animals for in vivo based studies, will be summarized. Finally, existing limitations are discussed, and future developments of the field are highlighted.

## In vitro culture methods and applications for cancer research

### Conventional ECM-drop culture

#### Animal-derived tumoroids in cancer research and their application

As tissue samples from animals often are easier accessible than most human biopsies, and as a big variety of murine cancer models exist, animal-derived tumoroids can be especially useful when human-derived tumoroids are not available. Therefore, many murine and other animal-derived 3D models have been established and used across all fields of cancer research (Table 1). Importantly, the availability of normal tissue samples and thus healthy organoid lines from animal models allows for the evaluation of adverse side effects of cancer therapies on healthy tissue [52].

**Table 1:** Summary of animal-derived tumoroid models, including murine, canine, and porcine 3D models for the most commonly diagnosed cancer types

Species	Cancer Type	Year	References
Mouse	Breast Cancer	2023 2022 2020 2016	[53, 54] [55] [56] [57]
	Lung Cancer	2021 2020 2018 2017	[58] [59, 60] [61] [62]
	Colorectal Cancer	2022 2020	[63–65] [66, 67]
	Prostate Cancer	2022 2019 2018 2016 2015 2014	[68] [69] [70] [71] [72] [73, 74]
	Gastric Cancer	2022 2021 2019 2014	[75] [76] [34, 77] [78]
	Liver Cancer	2021 2020 2019	[79] [80] [81, 82]
Dog	Breast Cancer	2022 2017	[83] [84]
	Lung Cancer	2023	[85]
	Colorectal Cancer	2019	[86]
	Prostate Cancer	2017	[87]
	Bladder Cancer	2020 2019	[88] [89]
	Thyroid Cancer	2023 2021	[90] [91]
Pig	Intestinal Cancer	2017	[92]

In the past, cancer research mainly relied on genetically modified mouse models (GEMMs) targeting genes commonly mutated in human cancers [93]. Thus, a variety of organoids and tumoroids from these mouse models exist, which can further be easily genetically modified in vitro. Two recent studies focused on the development of preclinical 3D models for ovarian cancer based on murine cancer models [94, 95]. The knock-out of ovarian cancer-specific genes was induced using either a Cre recombinase-based system or CRISPR/Cas9 in organoids derived from healthy murine tissue in vitro, leading to their malignant transformation. The group of Zhang et al. hereby focused on the influence of the mutational heterogeneity on treatment response, and showed that both tumoroids and mice with the same mutational background can be treated successfully with identical combination therapies [94]. The group of Löhmussaar et al. established a tumor progression model for high-grade serous ovarian carcinoma from two different epithelial lineages of mouse ovaries. Their findings support the dual-origin hypothesis of these tumors and confirmed lineage-dependent differences in drug sensitivities [95]. Thus, these studies highlight how murine tumoroids can be used as a model for identifying novel genotype-informed treatments, and for investigating the differences and vulnerabilities of tumors arising from distinct cell types, leading to more patient-specific and efficient therapy options.

Another approach using tumoroids derived from a prostate cancer (PCa)-specific mouse model was reported by Chan et al., who investigated genetic driver mutations of treatment-induced transformation and plasticity [68]. The group induced malignant transformation of healthy organoids by knocking out the PCa-specific genes *Pten, Rb1, and Tp53* in vitro and showed that over time the resulting tumoroids acquired an intermediate luminal-basal phenotype. An epithelial to mesenchymal (EMT) signature was further supporting tumoroid plasticity, a phenomenon that has recently also been investigated in pancreatic cancer (PaCa) organoids [96]. Of note, the authors reported that the PCa tumoroid plasticity was further induced by androgen ablation (the suppression or blockage of the androgen pathway using chemical compounds), the most common treatment for PCa, and identified JAK/STAT and FGFR signaling as the main drivers [68]. Since the inhibition of these two pathways converted the tumoroids back to an androgen ablation-sensitive more luminal phenotype, PCa patients may benefit from the same treatment. The researchers also tried to confirm their results in human PCa tumoroids. However, it is difficult to maintain human prostate organoids and tumoroids in long-term cultures, and the results did not overlap [68, 97, 98]. More studies are needed to verify these findings, but PCa is a good example for how animal-derived 3D models could be used as an alternative model for human cancer.

To investigate metastasis formation in addition to tumor heterogeneity, two of the main factors for poor overall survival of breast cancer (BrCa) patients, tumoroids from the C3(1)-TAg BrCa mouse model were used [54]. Individual tumoroid lines established from the same primary tumor showed heterogeneous invasive mechanisms that were classified into collective invasion or dissemination of single cells. Interestingly, KRAS expression was required for both mechanisms, and ERK inhibition blocked both invasive processes [54]. Additionally, it was shown that collective migration of BrCa cells is mediated by a keratin 14 and cadherin 3-positive subpopulation of leader cells [53]. These cells have an enhanced protrusive activity and interact with the TME to initiate invasion, and could become a novel target for preventing or treating BrCa progression. However, murine 3D cell models can not only be used to test the effectiveness of drugs, but also of other anti-cancer treatments like radiation. After irradiating murine intestinal organoid models, Du et al. demonstrated that the induction of inflammation with Zymosan-A promoted the regeneration of intestinal stem cells by up-regulation of ASCL2 [52]. Thus, Zymosan-A may be an effective radio-protective drug for the prevention of harmful side effects on surrounding healthy tissue and treatment of ionizing-radiation-induced intestinal injury.

In general, the results obtained using murine organoid systems that mimic human disease adequately reflect human physiology and mechanisms [52–54, 68, 94, 95]. However, rodents and mammals are inherently different, which can lead to unexpected discrepancies during translation to the clinic [99, 100]. Recently, the use of organoids and tumoroids derived from farm or companion animals has become more popular in the field of cancer research, as animals like cattle, horses, pigs, monkeys, dogs, and cats have a more similar anatomy to humans [101]. One big difference to rodents is that these animals can develop spontaneous cancer lesions, which makes them more suitable to study the mechanisms of cancer initiation and metastasis [83]. For example, in dogs bladder cancer (BlCa) arises spontaneously with similar pathology and genetics to human disease [89], while murine tumoroids do not fully reflect the characteristics of human BlCa [102]. Therefore, Elbadawy et al. focused on canine tumoroids derived from CTCs from urine samples to generate a more representative model to study BlCa [89]. Later the same group also established canine healthy bladder organoids to study the transformation of healthy cells to cancer cells [102]. The canine BlCa 3D models were then used to show that trametinib, a drug mainly used for melanoma treatment [103], and extracts from the Chaga mushroom [104], could be a potential therapy option for patients with BlCa. Recently, the same researchers also established canine healthy lung and lung cancer 3D models that resemble human disease as a new model for molecular analysis and drug testing of lung cancer [85]. It can be expected that additional organoid systems will be established from non-conventional animals in the near future, some of which might have high translational impact for human cancer [105, 106].

#### Human-derived tumoroids in cancer research and their application

Patient-derived tumoroids (PDTs) maintain the characteristics of the primary tumor and can thus be used as a model to identify the most efficient cancer treatment for individual patients, laying the foundation for personalized medicine [107]. Therefore, tremendous efforts have been made to generate organoids and tumoroids from diverse tissues and tumor types. Until now, PDTs from more than 20 different carcinoma types, including BrCa, lung cancer, and colorectal cancer (CRC) have been established (Table 2).

**Table 2:** Summary of human tumoroid models for the most commonly diagnosed cancer types

Cancer type	Year	Reference
Breast cancer	2022 2020 2018	[108] [109] [110]
Lung cancer	2019 2017	[24, 111] [112]
Colorectal cancer	2022 2016 2011	[113] [114] [11]
Prostate cancer	2022 2021 2014	[97] [115] [26]
Gastric cancer	2018	[116–118]
Liver cancer	2018 2017	[19] [119]
Cervical cancer	2022 2021	[120] [121]
Esophageal cancer	2021 2019 2018	[122] [123] [124]
Thyroid cancer	2023 2022 2021	[125] [126] [127]
Bladder cancer	2023 2018	[128] [129]
Pancreatic cancer	2018 2017 2015	[130] [112] [131, 132]
Endometrial cancer	2023 2019 2017	[133] [134] [112]
Ovarian cancer	2020 2019	[135, 136] [23]
Glioblastoma	2020 2019 2016	[137] [138] [139]
Head and neck squamous cell carcinoma	2023 2019 2018	[140] [141] [142]
Mesothelioma	2018	[143]
Renal cancer	2022 2021 2019	[144] [145] [146]
Merkel cell carcinoma	2022	[147]

Due to their advantages over 2D cell culture, PDTs are widely used for drug screening and therapy response prediction of patients [17]. Additionally, as it was shown that both animal- and human-derived tumoroids do not acquire additional mutations during long-term culture in vitro [148], numerous studies have already proven the usefulness of such 3D systems for precision medicine including CRC [113, 149–151], PaCa [152–154], as well as rare cancer types such as Merkel cell carcinoma [147]. In CRC PDTs, the response of patients to oxaliplatin was predicted with a sensitivity of 70% and a specificity of 71% [155]. Additionally, transcriptome analysis revealed that differences in oxaliplatin response were mediated by distinct genetic features, and 18 specific genetic alterations were identified as a potential biomarker panel for oxaliplatin resistance in CRC patients to aid clinical decision making. Another study focusing on the resistance of PaCa patients to neoadjuvant chemotherapy confirmed a high heterogeneity in patient responses, supporting the notion that personalized medicine approaches might be more efficient [156]. Hennig et al. hypothesized that the observed resistance is mediated by resistant clones residing in the tumor that get enriched under systemic treatment. In addition to intrinsic resistance of tumors, acquired resistance can also hinder efficient treatment. Most research on the underlying mechanisms of this phenomenon have been generated using 2D cell lines, but recently resistant tumoroid models have been established as a more representative system [157–159].

As confirmed by the recent improvements of transcriptome and proteome analysis on single cell level, one of the remaining problems for efficient cancer treatment is the intra-tumoral and inter-patient heterogeneity [160, 161]. Studies showed that between different regions of the same tumor, drugs can have varying inhibitory effects [162, 163]. This highlights the fact that biopsies might not be representative for the drug response of the whole tumor, and that specific combinatorial therapies might be necessary to target all tumor subclones [164, 165]. To analyze whether PDTs can reflect the intra-tumoral heterogeneity of a primary tumor, De Witte et al. compared the drug responses of ovarian cancer PDTs derived from different sites of the same primary tumor for a small patient cohort [162]. The group reported that individual tumoroids showed a differential response of 31%, defined as a more than 10-fold change in IC50 value, to the same treatment, proving inter-patient heterogeneity. Additionally, individual tumoroids from the same primary tumor showed high differences in drug response in six out of seven cases, suggesting that PDTs genetically maintain the heterogeneity and drug sensitivity of the original tumor [162]. Interestingly, it was recently shown that a more physiological culture medium changes the drug response of ovarian tumoroids compared to commonly used organoid medium, highlighting the necessity for careful selection of experimental conditions [166]. Using a similar approach, the intra-tumoral drug response differences of liver cancer were investigated [163]. In this study, 27 PDTs were generated from different areas of five primary lesions and a total of 129 cancer drugs were tested. Even though a high inter-patient and intra-tumoral heterogeneity was confirmed, the researchers identified several pan-effective drugs that showed an inhibitory effect for most of the tested tumoroids [163].

The high heterogeneity of cancer is a significant problem for efficient treatment; however, metastasis formation is still the main reason for cancer related death. To elucidate the differential drug responses between primary tumors and matched liver metastases of CRC, Mo et al. generated tumoroids from both samples of 25 patients, and showed that the intra- and inter-patient heterogeneity is reflected by the in vitro 3D models [167]. Interestingly, although transcriptomic analysis revealed differences between primary and metastatic tumoroids, drug sensitivities were highly consistent. Thus, the authors suggested that the response of metastatic lesions to specific drugs could be predicted using tumoroids derived from primary tissue for a personalized medicine approach [167].

The predictive potential of PDTs and their translation to the clinics has also been investigated for various other cancer types, such as PaCa [168], and brain cancer [169], two highly aggressive cancer types with limited or inefficient treatment options. In a translational approach, tumoroids of different stages of PaCa and brain tumors were used to predict the optimal treatment for each patient in a clinically relevant time frame [168, 169]. Even though both studies confirmed that tumoroids reflect inter-patient heterogeneity and patient-specific drug responses, limitations of varying efficiencies of PDT generation, time management, but also cost, have to be overcome for direct translation of tumoroid research to the clinics. Especially for rare cancers, where a major problem for efficient treatment is the lack of prognostic and diagnostic biomarkers due to small patient numbers [170, 171], tumoroids might be suitable to improve treatment response and survival rates of patients [172]. For example, PDTs of PD-L1 negative mucinous adenocarcinoma of the appendix were used to predict response to chemotherapeutic drugs and targeted therapy for one individual patient [173]. PDT drug response correlated well with the patient response, and the tyrosine kinase inhibitor dasatinib was identified as a possible treatment option for this patient.

In addition to evaluating the efficiency of commonly used treatments for patient-specific precision medicine, PDTs can also be used to test the efficacy of novel treatment regimes. As an example, lung cancer PDTs are highly sensitive to the tyrosine kinase inhibitors dabrafenib and trametinib, which are commonly used for melanoma treatment, suggesting these drugs as a novel therapy for lung cancer [174].

In summary, several studies have provided clinically relevant evidence that PDTs can be a suitable model for a precision medicine approach to overcome the problem of high heterogeneity in drug responses between patients. In line with this, PDTs have also been used to identify pre-existing and acquired resistance to commonly used drugs, to avoid treatment of patients with inefficient therapies.

PDTs are not only suitable to predict the response of tumor cells to chemotherapy or targeted drugs, but also to other treatments like radiotherapy [141, 175–178]. Radiation directly leads to the induction of single-strand and double-strand breaks in DNA, as well as the generation of reactive oxygen species and the upregulation of oxidative stress signaling pathways, and is thus commonly used as a combinatorial treatment with chemotherapy [179]. For patients with locally advanced rectal cancer, the standard treatment is neoadjuvant chemoradiation followed by total mesorectal excision of the tumor [175]. Drug response of rectal PDTs matched chemoradiation responses in patients with an accuracy of up to 84% [175, 176], and a sensitivity of 78.01% with 91.97% specificity [176]. The predictive potential of PDTs for radiotherapy has also been confirmed for head and neck squamous cell carcinoma (HNSCC), where the combination of the EGFR inhibitor cetuximab and radiotherapy resulted in increased cell death in vitro, and showed higher efficacy compared to radiotherapy alone [141]. A recent study from the same researchers confirmed the predictive potential of HNSCC tumoroids by correlating tumoroid therapy response with patient clinical response to model the efficacy of chemoradiation for these patients, and the further use for biomarker selection and validation [177]. In addition, PDTs offer an advanced model to study the dynamics and mechanisms of radioresistance. Glioblastoma PDTs reflected in vivo resistance mechanisms mediated by treatment-induced senescence to combination treatment with temozolomide and radiation, suggesting the use of PDTs for studying the underlying mechanisms of drug resistance [178].

### Co-cultures and advanced culturing systems

**Fig. 2:**
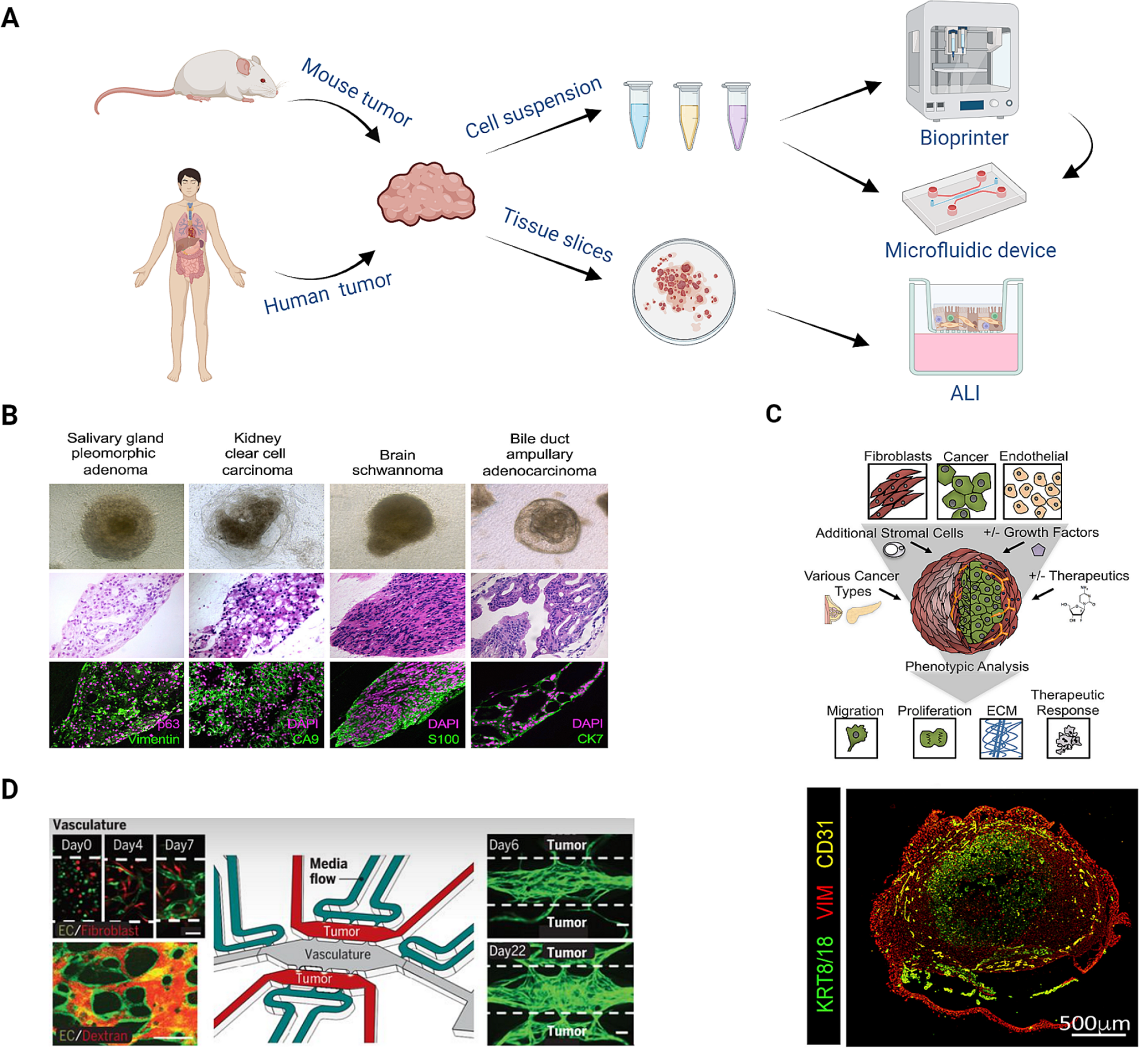
Co-culture techniques for tumoroid models. (**A**) Overview of state-of-the-art co-culture techniques in cancer research. (**B**) ALI tumoroid models of different cancer types for TME studies on day 30. Phase contrast (top), H&E (middle), and immunofluorescent staining (bottom) of ALI PDTs for DAPI (purple) and VIM, CA9, S100 or CK7, respectively (green) [183]. (**C**) Schematic depicting the generation of a bioprinted tumoroid co-culture model including fibroblasts and endothelial cells (top) [184]. Immunofluorescent staining of sections of bioprinted tumoroids stained for CD31 (yellow), VIM (red) and KRT8/18 (green) (bottom) [185]. (**D**) Schematic (middle) and immunofluorescence stainings (left, right) of a vascularized BrCa tumoroid-on-a-chip model for studying the TME and drug sensibility of cancer cells [186]. B. Copyright Elsevier, C. Copyright Wiley-VCH GmbH, D. Copyright Royal Society of Chemistry. Reproduced with permission. Created with BioRender.com

Recent advances in the field of cancer research highlight the critical importance of not only the tumor itself but also its surrounding TME. The interplay between the tumor and the TME is a key determinant in tumor growth and progression, making it an essential focus for comprehensive studies [180–182]. Traditional 2D or 3D cell culture models are limited in their ability to fully capture these complex interactions, prompting the development of more sophisticated methods. In recent years, three innovative approaches have emerged to model the TME in a more physiologically relevant manner. This chapter will discuss: (1) the integration of co-culture techniques with an air-liquid interface (ALI), which allows for relevant interactions between tumor cells and their environment; (2) the application of bioprinting to create tumoroids including cells of the TME, which offer a more realistic, 3D structure for studying tumor behavior; 3) the incorporation of microfluidic channels, which can be used independently or combined with bioprinting, to simulate dynamic factors like blood flow or targeted drug administration (Fig. 2A).

#### Co-cultures

To investigate the interplay between cancer cells and their surrounding environment, advanced co-culture models, including the culturing of tumoroids with cancer-associated fibroblasts (CAFs) [187, 188], lymphocytes [189], and myeloid cells such as macrophages [190–192], or even bacteria [193] have been used. Indirect cell interactions can be mimicked by the addition of cell-conditioned media, which contains secreted molecules, to another cell type. However, this co-culture system only allows for single-directional response, which can be overcome by the use of transwell cultures. Individual cell types are separated *via* a mesh leading to diffusion of secreted molecules and therefore to a multi-directional interaction [13, 188]. However, as direct cell-cell contact is essential for various physiological processes, indirect co-culture systems can only be used to investigate the effect of metabolites, cytokines, and other secreted molecules. In direct co-culture systems different cell types are embedded in the same ECM and then either submerged in culture medium or maintained as an ALI. Because of the direct cell-cell contact, different aspects of the interaction between the tumor and its TME can be studied. However, as it is more complex to isolate specific cell types for downstream analysis, single cell read outs or optical techniques are required to efficiently analyze cell-cell dependencies or their crosstalk [187, 190, 194, 195]. As important factors normally found in the TME are lost upon dissociation of the tumor tissue and the subsequent artificial assembly of the isolated cells for direct co-culture systems, Ootani et al. established a method to culture cells in their original environment by preserving the TME using ALI models [196]. Dissociated tissue is hereby directly embedded into an ECM such as collagen type I and/or a hydrogel, seeded onto a transwell mesh, and then cultured in a way that only the basal part of the ECM or tissue is in contact with the culturing medium while the other part is exposed to the atmosphere [183, 188] (Fig. 2A). ALI models have already been established for several cancer types, such as salivary gland cancer and kidney cancer (Fig. 2B). This method leads to the differentiation and maturation of the cell types present in the tissue, resulting in organoids or tumoroids containing epithelial cells, as well as stromal and immune cells [13, 183, 197]. This way, PDTs can be cultured for months while mostly preserving the original TME, thus reflecting the physiological tumor site. However, the limited availability of primary tumor tissue, the slow growth of a tissue ALI culture, and the partial loss of stromal cells during long-term culture, makes this method unsuitable for high-throughput analyses such as drug screens [198]. Tissue ALI culture can nevertheless be a powerful direct co-culture model for studying processes like tumor progression, therapy response to selected drugs, or the presence and infiltration of immune cells in the original tumor tissue.

##### Stromal cells

As mentioned above, stromal cells in the TME are involved in many tumor promoting processes. CAFs can accelerate tumor growth by synthesizing and remodeling the ECM, and by producing growth factors that induce angiogenesis. Additionally, by negatively affecting drug access to the tumor they can influence therapy response [187, 199–201]. We recently developed a PDT fibroblast co-culture model using matched tumoroids and CAFs or normal fibroblasts (NFs) isolated from CRC patients [188]. Both NFs and CAFs were able to support the growth and differentiation of tumoroids without the addition of specific niche factors, suggesting a better reflection of the in vivo tumor heterogeneity than conventional PDT cultures. In addition, we observed an enhanced cancer-promoting phenotype of NFs upon co-culture with the tumoroids, highlighting the dynamic and interdependent cross-talk between the tumor and its microenvironment [188]. These co-culture models not only reflect the microenvironment but can also be used to model the influence of the TME on cancer cell drug response. By testing the response of both human and murine liver tumoroids to conventional anti-cancer drugs such as regorafenib, sorafenib, and 5-FU, it was shown that the direct co-culture with CAFs or the addition of CAF-conditioned medium to the liver tumoroids led to a higher resistance of the cancer cells [79]. Also, PaCa PDTs, which were directly co-cultured with CAFs, showed higher resistance against commonly used PaCa drugs when compared to tumoroid-only cultures [187]. Single cell RNA-Sequencing revealed differences in gene expression in the PDTs induced by the CAFs, which led to the induction of EMT in the cancer cells. The authors suggested that this EMT induction contributed to the chemo-protectant effect seen in their co-culture system [187]. Similar EMT-inducing effects have been seen in other studies where EMT was partially induced in cancer cells by direct co-culturing of CAFs and human CRC tumoroids mimicking either early or late stage CRC [202, 203]. Further on, inhibition of WNT signaling in CAFs was shown to induce different CAF subtypes resulting in repression of EMT in CRC tumoroids, highlighting how stroma cells could be targeted for anti-cancer therapies [204].

The importance of specific fibroblast subtypes and the synergistic cross-talk between the tumor and its TME for cancer progression is well understood. However, how tumor cells reprogram NFs into CAFs is still largely unknown. To investigate whether the mutational status of a tumor could reprogram and determine the fibroblast phenotype, Shaashua et al. analyzed the differences between NFs indirectly co-cultured with either *BRCA* wild-type or germline *BRCA* mutated PaCa tumoroids [205]. Different fibroblast subtypes were indeed derived from the NFs depending on the *BRCA* mutational status, thus suggesting that each tumor might shape its individual TME.

In summary, germline mutations can not only change the behavior of the tumor itself but can also influence the surrounding stromal cells [205]. Thus, to overcome stroma-mediated resistance, therapies have to target multiple cell types in the TME simultaneously, and personalized medicine approaches are necessary for efficient treatment.

##### Leukocytes

Immune cells are another important cell type in the TME inducing both anti- and pro-tumorigenic effects, which represent promising targets for efficient cancer treatment. Even though co-cultures of immortalized 2D cell lines with immune cells, or animal models, already led to the development of promising cancer treatments targeting the immune system, tumoroids co-cultured with immune cells might be another suitable model for testing patient-specific responses to various immunotherapies [32, 40, 206, 207].

Many groups have already established co-cultures of tumoroids with lymphocytes focusing on T cells and natural killer (NK) cells to improve the modeling of immunotherapies like CAR T cells and immune checkpoint blockade [208]. CAR T cell therapy has proven successful for leukemia, but models for investigating the cytotoxic effect of these cells on solid tumors like CRC are still needed. Therefore, Schnalzger et al. developed a CRC PDT co-culture model as a sensitive in vitro platform to study patient-specific treatment responses [207]. Other groups focused on investigating the mechanism of PD-1/PD-L1 immune checkpoint blockade using advanced co-culture models, and showed that human and murine lymphoma tumors and their TME could be cultivated for several weeks using an adapted ALI model [183, 209]. The system reflected the physiological properties of the tissue, and cell composition including lymphoid cells and supporting T helper cells was preserved. Importantly, the ALI cultures functionally recapitulated PD-1/PD-L1 dependent immune checkpoint blockade, highlighting the potential of this model to develop personalized immunotherapies for lymphomas [183, 209].

Similarly, a recent human tumoroid and immune cell co-culture model for gastric cancer was developed [189]. In this model, dendritic cells were first activated by a patient-derived tumor antigen, and then used to prime CD8 + T cells. The authors suggest that the direct T cell activation in their model is superior to activation by anti-CD28-coated plates, which was used in a similar co-culture model [210].

Moreover, focusing on immune checkpoint blockade therapies, novel bispecific antibodies for PD-1 and PD-L1 were used to restore NK and CD8 + T cell activity in ovarian PDT co-cultures with intra-tumoral immune cells [211]. Immune cell reactivation was shown to be mediated by the partial downregulation of BRD1, and inhibition of BRD1 had a similar anti-tumoral effect in vitro and in vivo. Thus, BRD1 inhibition could provide a new therapy option for ovarian cancer patients to circumvent immune cell evasion [211].

Myeloid immune cells such as tumor-associated macrophages (TAMs) play an important role in the TME to support tumor growth, and thus represent important therapeutic targets. Therefore, both conventional organoid [212] and ALI co-culture models combining tumoroids and macrophages have been developed [213].

By including multiple immune cell types in one co-culture model, it is possible to further analyze the complex interplay between the tumor, and between different kinds of immune cells. This way, it was shown that reprogramming of TAMs following monoamine oxidase A inhibitor (MAO-Ai) treatment, a widely used class of drugs for the treatment of depression and Parkinson’s disease, enabled the induction of anti-tumor T cell reactivity in a melanoma co-culture model [214]. MAO-Ai could thus be used in combination with PD-1/PD-L1 inhibitors to reactivate the anti-tumor immune response as a new potential treatment for several cancer types.

However, co-cultures can not only be used to study the interactions between tumoroids and multiple immune cells but also to analyze more complex interactions between cells in the TME, including tumor cells, immune cells, and stromal cells. For this, Sufi et al. developed the Thiol-reactive Organoid Barcoding in situ Mass Cytometry (TOBis MC) protocol, which allows for the analysis of organoid and tumoroid lines in co-culture with leukocytes and fibroblasts on single cell level to study the interactions between TAMs and CAFs in high-resolution [215].

In summary, organoid and tumoroid models in co-culture with other cell types can be used to model the complex interactions of the TME. Even though the benefit of these co-culture models for improving or finding novel treatment options has been proven, the assembly of these models is time consuming, and high-throughput methods still have to be improved. Automated methods like bioprinting could help to overcome some of these limitations.

#### Bioprinting

Bioprinting allows for the exact positioning of different cell types and biomaterials in a 3D space with the help of a mechanical and computer-assisted system to mimic the in vivo spatial architecture of a tissue or tumor and its microenvironment [216]. The biggest advantage of using a bioprinting-based approach to generate preclinical cancer models is the standardization of cell dispensing [217], and the possibility to construct an artificial 3D tumor including different cell types, structures, and ECMs for more precise personalized medicine approaches [218].

Early studies focused on generating such models by using bioprinted cancer cell lines or single cell suspensions [184]. Recently, the dispersion of organoids and tumoroids together with stromal cells allowed for the development of co-culture models including the TME (Fig. 2C) [219].

CRC microtissues were produced according to patient-specific colonoscopy images by printing PDTs surrounded by healthy organoids to model the interaction of a tumor with normal adjacent tissue [220]. As the in vitro treatment response of these microtissues to the standard 5-FU therapy reflected the patient response, the model could be used as a more physiological drug screening platform. Additionally, the patient-specific risk of tumor invasion into the surrounding tissues was calculated in this model by correlating the number and distance of invading tumor cells. This could provide a real-time quantitative readout for analyzing cancer progression and metastasis [220].

In another recent study, microtissues consisting of patient-derived lung tumoroids in co-culture with matched CAFs and endothelial cells were generated [221]. After printing the vessel structures and seeding the CAFs, the tumoroids suspended in a hydrogel derived from porcine lung tissue were printed into the same compartment. An active fusion of the stromal cells and tumoroids was observed, and microvessels formed to directly interact with the other cell types. After administering the drug poziotinib through the vessel structures it was seen that both the endothelial cells and CAFs, but also the CAF-secreted matrix, protected the lung tumoroids from the treatment. Thus, this model could be used to further test the influence of cell-cell or cell-matrix interactions on the efficacy of drug delivery to the tumor tissue [221].

Apart from printing models mimicking the TME, bioprinting can be used for standardized cell dispensing for high-throughput applications. However, one drawback is the difficulty of printing bioink into small wells as the utilized ECMs can spread in the well and thus do not maintain the shape necessary for 3D cell growth. To circumvent the spreading, patient-derived glioblastoma or sarcoma cells were mixed with a bioink consisting of hyaluronic acid as well as collagen, and then printed into wells coated with gelatin, which was later removed and substituted with medium [222]. In addition, acoustic bioprinting of small droplets onto a hydrophobic substrate was used to generate BlCa-derived tumoroids consisting of both cancer cells and CAFs [223]. This method allows for the generation of large numbers of uniform tumoroids that mimic the TME, which can easily be dispensed into small wells and be used in high-throughput drug screens for personalized therapy.

Thus, the combination of 3D cell models and bioprinting could help to facilitate reproducible drug testing, and the investigation of cellular processes in a 3D space with multiple cell types and structures. However, these printed models are still static and do not reflect the effect of mechanical forces like dynamic flow, or chemical gradients, which influence the tumor cell phenotype in vivo [224].

#### Fluidic devices

The important physical and chemical characteristics of the TME can be modeled with microfluidic systems, where cells are cultured in chambers connected to microchannels supplying oxygen, growth factors, or drugs *via* dynamic perfusion [225–227] (Fig. 2D). In a proof of concept study, CRC PDTs cultured on fluidic devices showed a higher viability, proliferation rate, and tumoroid formation efficiency, compared to tumoroids cultured in static ECM drops on a plate [228]. Additionally, chip-grown tumoroids were bigger in size and showed a multilayered morphology with crypt-like projections similar to the human colon, while the conventional tumoroids formed more cystic monocellular structures. Interestingly, when testing the response of PDTs to 5-FU no significant differences were seen between the culturing methods, suggesting that chip-grown 3D cells maintain their phenotype, while having a growth advantage, and thus validating the use of fluidic devices for tumoroid culture and disease modeling [228].

However, the tumor niche consists of multiple cell types, and an extensive vasculature, through which nutrients, oxygen or drugs get delivered to the cancer cells. In order to mimic this vascular transport of factors and metabolites to the tumor, endothelial cells together with fibroblasts were grown in microfluidic chambers to produce a 3D microvascular network, before adding primary BrCa tumoroid-like structures into a separate adjacent compartment [186]. Using this setup, vessel outgrowth towards the tumor cell chamber was observed in addition to tumor cells invading the vasculature chamber after inducing EMT *via* TGF-β treatment. Aside from its suitability for physiological drug testing, this chip design could be a useful tool to study and easily visualize differences in angiogenesis, proliferation, and migration [186]. Importantly, it has already been shown before that fluidic devices provide a more physiological model to study cancer cell migration [229].

Using microfluidic devices with tumoroids alone, recent papers already described the advantages of chip-based models for a personalized medicine approach and more representative drug testing compared to static models. Both Mazzocchi et al. [143] and Jung et al. [230] used PDTs to perform medium-throughput drug screens for mesothelioma or small cell lung cancer (SCLC) patients, respectively. Drug response to specific chemotherapeutic therapies was different between the tumoroid lines with different mutational backgrounds but correlated well with the patient response. Importantly, SCLC tumoroids on the chip maintained a chemotherapy resistant core, which might be due to insufficient drug delivery also observed in in vivo tumors [230].

To facilitate the translation of this drug screening method to the clinics, Schuster et al. developed an automated high-throughput screening platform on a chip [231]. The authors constructed a microfluidic system with which 20 independent experimental conditions could be tested on up to 10 different patient-derived 3D cell lines. A customizable software allows for the stimulation, assaying, and imaging of the tumoroids without human intervention. Interestingly, combinatorial treatments and time-dependent administration with multiple rounds of treatment showed greater therapy efficacy on PaCa tumoroids compared to single treatments [231].

To investigate the influence of the tumor stroma and the method of drug delivery on treatment efficiency, a fluidic system mimicking the TME for drug testing on PaCa PDTs has recently been established [232]. The vasculature was modeled by growing endothelial cells in a perfusable microfluidic scaffold. Tumoroids co-cultured with fibroblasts, which resulted in increased proliferation of cancer cells and enhanced tissue stiffness, were then added into adjacent compartments. When administering the chemotherapeutic drug gemcitabine to the co-culture *via* diffusion through the vessel-like structures, the drug had a reduced efficiency compared to the administration to tumoroid single cultures or to static co-cultures. Thus, the authors were able to recapitulate the microenvironment of a vascularized pancreatic tumor and to demonstrate the inhibitory effect of the vasculature cell barrier and the stroma on drug efficiency [232].

Interestingly, another PaCa-on-a-Chip model was used to show that stroma-targeting agents do not influence the cell viability of tumoroids in monoculture, but lead to a significant increase of chemotherapeutic anti-cancer effects in a fluidic device co-culture [233]. Using a similar model, it was reported that different drugs show different efficacies in targeting PaCa cells in normoxic versus hypoxic conditions [234]. Thus, both publications highlight the importance of including cells of the TME and relevant oxygen levels for microfluidic drug response studies.

In conclusion, tumoroid-on-a-chip models provide a powerful tool to investigate biological processes and to assess drug responses in an efficient and more physiological manner compared to static tumoroid cultures.

## In vivo methods and applications

### Modeling of tumor biology

**Fig. 3:**
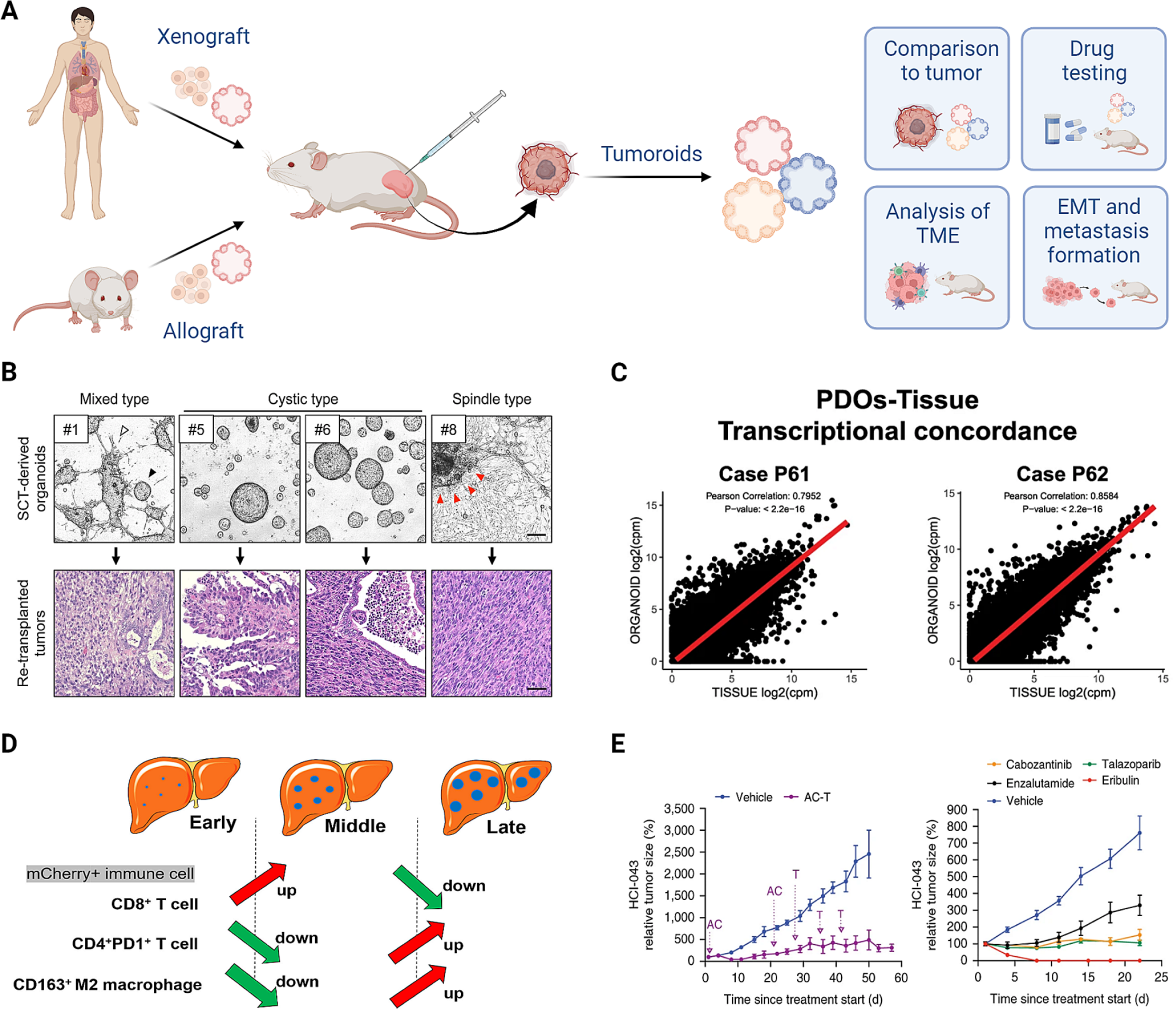
In vivo methods for tumoroid models. (**A**) Overview of in vivo applications of both human and murine tumoroids. (**B**) Light microscopy pictures of tumoroids with different phenotypes (Scale bar 200 μm) and H&E staining of corresponding tumors after re-inoculation of subcutaneous tumor-derived tumoroids (Scale bar 50 μm) [237]. (**C**) Pearson correlation coefficient plots of gene expression between prostate tumors and matched tumoroid lines [238]. (**D**) Dynamic changes of immune cell populations during early, middle, and late stages in a novel tumoroid-based liver metastasis model [239]. (**E**) Real-time treatment of patient-derived xenograft mice representing a pretreatment tumor with either patient-matched neoadjuvant therapy (includes the drugs doxorubicin hydrochloride (Adriamycin) and cyclophosphamide, followed by treatment with paclitaxel (Taxol) = AC-T) (left) or with drugs selected from a xenograft-derived tumoroid screen (right) [108]. B, C, E adapted from corresponding citations; Springer Nature (Creative Commons Attribution 4.0 International). D adapted from Wiley (Creative Commons Attribution 4.0 International). Created with BioRender.com

Even though tumoroid models can deliver meaningful translatable results for basic cancer research and patient therapy options, the assessment of systemic effects and the influence of a more complex in vivo TME can only be validated in animal models [51] (Fig. 3A). In the past, whole tissue pieces, digested primary tissue, or 2D cell lines were grafted into animals as allografts (between same species) or xenografts (between different species) either subcutaneously or orthotopically [235]. These models have lately been expanded to transplant organoid and tumoroid lines into animals as allografts and xenografts. Importantly, the pathological and invasive features of the original tumor and drug response of the patient are retained in these models, providing a suitable alternative to investigate mechanisms of malignant transformation, tumor plasticity, and metastasis in vivo [236].

#### Cancer initiation and progression

To study the tumorigenic potential of cancer cells based on common mutational patterns specific for endometrial cancer, murine tumoroids that overexpress *Kras*^*G12D*^ and carry genetic deletions of various tumor suppressor genes (*Pten*, *Tp53*) were allografted subcutaneously into nude mice [237]. Different mutational backgrounds led to the development of specific tumor subtypes with corresponding tumoroids having an either more cystic or spindle-like morphology because of an irreversible EMT during transformation. This also correlated with a less or more aggressive phenotype, respectively [237] (Fig. 3B).

As the specific cancer subtype also plays an important role in the aggressiveness and treatment of PaCa, Miyabayashi et al. established an in vivo model to investigate the role of patient-specific genetic and epigenetic aberrations for PaCa heterogeneity [240]. The authors derived tumoroids from patients and xenografted them orthotopically into the pancreatic ducts of immunodeficient mice. Tumors were either indolent with a luminal morphology or progressed to a more aggressive basal-like subtype based on epigenetic aberrations and deregulation of KRAS. In contrast to Maru et al. [237], this group argued that cellular plasticity is mainly influenced by the stroma and microenvironment rather than cellular clonality, thus suggesting that allo- and xenografts should be performed orthotopically to recapitulate the corresponding tumor niche for translatable results [240].

#### Cellular plasticity and metastasis

Cellular plasticity and clonality can not only be influenced by the microenvironment, but also by specific therapies [241]. To investigate resistance mechanisms of androgen pathway directed therapy, Lee et al. generated tumoroids from patient-derived xenograft models of bone metastatic PCa. Androgen ablation led to the development of reversibly dormant basal-luminal-like hybrid cells, highlighting how some cancer therapies might even support the progression of the disease, thus suggesting that treatment regimens might have to be reviewed [241]. Apart from tumor heterogeneity and plasticity hindering efficient treatment, metastasis formation is one of the leading causes of cancer-related deaths [242]. CTCs can spread to distant secondary sites to induce metastasis [243]. As CTCs are a rare and heterogeneous cell population, De Angelis et al. established a model to study the biological features and possible drug sensitivities of these cells [244]. The group derived tumoroids from both orthotopic CRC xenografts and CTCs isolated from the blood of the same mice. As expected, CTC-derived tumoroids showed a more aggressive and migratory phenotype in addition to higher stem cell and EMT marker expression compared to the xenograft-derived tumoroids. The authors also showed that CTC-derived tumoroids are highly sensitive to drugs targeting the Survivin pathway including YM155 and quercetin, providing a new possible treatment to inhibit metastasis formation. As this model reflected the properties of CTCs isolated from CRC patients, De Angelis et al. hypothesized that in the future patient-specific drug sensitivities could be studied in a metastasis xenograft model established from the patient’s primary tumor even before the onset of metastatic disease, and thus prevent tumor progression [244].

The same group also developed another preclinical metastasis model for CRC [245]. After orthotopically grafting patient-derived cells into immunodeficient mice, the authors generated tumoroids from the primary tumor site and from spontaneous liver metastases and analyzed the EMT state together with the drug response. As expected, metastatic tumoroids displayed a more mesenchymal phenotype and increased chemoresistance. Interestingly, high numbers of CTCs in patients correlated with an increased engraftment efficiency of the corresponding tumor-derived cells, again highlighting the important role of CTCs for cancer progression [245].

EMT is not only an important characteristic of CTCs, but also of the cancer cells in tumor lesions. Recently it was shown that hybrid cells, presenting both epithelial and mesenchymal characteristics, can be found in human tumor tissues, GEMMs, and tumoroids of BrCa [246]. The number of these cells correlated with worse overall survival in patients, while driving invasion in vitro. Using tail vein injection or orthotopic engraftment of tumor cells or tumoroids, respectively, heterogeneous EMT states were identified within the same tumor. Sequential molecular EMT programs were essential for tumor cell invasion and colonization during the metastatic process. Thus, both models could be used to further investigate the influence of the EMT status on patient survival [246].

To further understand the mechanism of metastasis formation, the role of the immune system in the metastatic niche was also recently investigated in a PCa model of liver metastasis [239]. Murine tumoroids generated from GEMMs with PCa specific mutations were orthotopically allografted into immunocompetent mice, which resulted in metastatic spread to the liver after only 30 days. This model is not only less time consuming compared to original GEMMs and xenografts, and more reliable than patient-derived cell lines, but importantly also allows for the investigation of the immune cells in the metastatic niche. The authors found that an immune suppressive microenvironment mediated mainly by neutrophils, anti-inflammatory macrophages, and CD4 + T cells was essential for the survival of tumor cells and progression of metastases (Fig. 3D). This model might be relevant for testing drugs targeting immune cells, and for elucidating cell-cell crosstalk in the metastatic niche [239].

In summary, organoids and tumoroids can be used in an in vivo setting to investigate many aspects of cancer development and progression, highlighting the advantages of orthotopic grafts and allografts. Thus, these models are highly suitable for drug testing and the development of novel therapies.

### Therapy development and drug testing in vivo

Patient-derived in vitro models in combination with matched in vivo models represent a suitable model system for personalized medicine applications. Multiple groups have used this approach to study cancer subtypes, patient-specific drug responses, and to identify potential alternative therapies. Importantly, while both 3D in vitro and in vivo models retain the genetic and epigenetic background of the primary tumor (Fig. 3C), xenograft-derived 2D cell lines can acquire additional mutations in culture, suggesting that 3D culture systems are more stable and representative models for drug screening [247].

Several recent studies provided evidence for the usability of PDTs for different tumor types, including endometrial cancer [248], glioma [247], BrCa [108], and metastatic PCa [238]. These “living” biobanks reflected patient-specific drug response, mimicked tumor metastasis [248], and were used to identify alternative treatment options such as the chemotherapeutic dianhydrogalactitol (VAL-083) for glioma [247], or multi-kinase inhibitors for therapy resistant metastatic PCa [238].

Additionally, Guillen et al. reported a first proof that results generated by patient-specific tumoroids in vitro could be translated to the clinic [108] (Fig. 3E). The group had already generated a xenograft model with a matched tumoroid line of a BrCa patient and performed a drug screen identifying eribulin as a novel drug that showed effective growth inhibition in vitro and complete regression with no recurrence in the mice. The patient went into complete remission after being treated with the same drug, however due to other complications later unfortunately died. Nonetheless, this case study showed that drug testing using xenografted PDTs is feasible in real time and can lead to beneficial results for patients [108]. Drug screens with extensive compound libraries can also help to further elucidate the mechanism of cancer progression. Even though many epigenetic aberrations play a role in the progression of BlCa, the use of epigenetic drugs as a potential treatment has so far only been investigated in five clinical trials [249]. The histone deacetylase activator SRT1720, which specifically targets SIRT1, was recently identified as a potent drug to inhibit the growth of both BlCa tumoroids and xenografts [249]. Mechanistically, SIRT1 activation resulted in deacetylation of HIF1α and subsequent repression of the hypoxia pathway. This pathway has been correlated with poor prognosis in patients, highlighting the potential of epigenetic therapies for BlCa [249]. In contrast, SIRT1 was also reported to promote the progression of BlCa [250, 251]. However, these studies were performed on 2D cell lines and might thus be less representative compared to 3D models and xenografts [249].

Apart from mouse models, rats are a widely used animal model for cancer research and treatment development. By orthotopically transplanting kidney tumoroids derived from patient-specific iPSCs into rats, a fully vascularized xenograft model for angiomyolipoma, a rare kidney tumor, was generated [252]. Using this model, the therapeutic efficacy of mTOR inhibition by rapamycin-loaded nanoparticles, the main treatment for angiomyolipoma, was confirmed, suggesting that this model would also be suitable to identify novel alternative treatments for this rare cancer type [252].

Additionally, zebrafish models are well established to study tumor metastasis and are also commonly used for in vivo drug testing [253]. A comparative analysis of relapsed pediatric malignancies used patient-derived spheroid cultures, tumor cells isolated from corresponding mouse xenografts, long-term organoid-like cultures, and zebrafish xenografts to compare drug responses in the different model systems. The zebrafish model proved to be a promising addition to current drug testing tools to rapidly assess potential treatments in vivo, and could be included for personalized medicine approaches in the future [253].

In summary, a variety of different animal- and patient-derived in vitro and in vivo models are available, which can be tailored to answer specific questions for basic and translational research.

## Limitations

Even though various studies have shown that PDTs are a reliable model system for the evaluation and discovery of anti-cancer drugs, several limitations have to be considered for tumoroid-based research.

Depending on the tumor type, only limited patient tissue material might be available. In addition, some ethical concerns regarding consent, ownership, and data integrity should be considered for the use and application of PDTs [254]. Also, the establishment success rates vary immensely between different tumor types, and whether PDTs are established from fresh or frozen tissue. This additionally negatively influences the translation to the clinics [255]. In general, establishment of 3D cell lines is more time- and labor-consuming compared to 2D cell lines. The different ECMs, and growth media containing specific niche factors, used for organoid and tumoroid culture do not adequately reflect the molecular features and stiffness of the primary TME, and one major drawback is the high variability in their composition. Additionally, ECMs are mostly derived from mice [256], which raises the issue of possible influence of cross species interactions, when culturing human-derived 3D cell lines in murine-derived ECMs [257, 258]. Another problem with 3D cell culture is the overgrowth of tumor cells by normal cells that were present in the tissue of origin. This is common in lung [259] and PCa [73] tumoroid cultures, presenting a limitation for cultivating pure tumoroid cultures over extended periods.

It has been shown that organoid and tumoroid models stably reflect inter-patient and intra-tumor heterogeneity [148, 260, 261]. However, tumoroid lines established from different sites of the same primary tumor show highly variable drug responses [162], raising the question whether sampling issues might limit the representative use of tumoroids for personalized drug screens. High-throughput screening using tumoroids as model systems is still limited by the fact that analysis methods are not standardized, and automation is needed for reliable high-throughput applications. Some commonly used readouts for these models are metabolic cell viability assays such as Cell Titer Blue, a resazurin-based fluorescent measurement [262], Cell Titer Glo (CTG) 3D, a luminescent-based agent that measures ATP content in spheroid [263, 264] and tumoroid screenings [219, 265, 266]. Phenotypic readouts can be performed on fluorescently-labelled or stained organoids and tumoroids either over a period of time [231, 267], or at a pre-defined endpoint [268, 269]. Using a multiparametric microscopy-based readout, more information up to single cell resolution can be acquired [270]. However, because of the size and 3D structure of organoids and tumoroids, Z-stacks have to be used to generate high-resolution imaging data; this is a time-consuming process that generates large amounts of data difficult to store and analyze [270, 271]. Additionally, computational analysis methods that are optimized for imaging of 3D structures with defined parameters for organoid and tumoroid phenotypes are needed [270, 272]. Compared to imaging analysis, metabolic assays like CTG 3D are less time consuming and the readout is easier to process and interpret [270]. However, less information can be obtained with this method as it is an endpoint-measurement representing the result of all cells in one well. Thus, a potential heterogeneity in drug response between the cells of a tumoroid cannot be analyzed [270]. Even though both metabolic and imaging methods are suitable for high-throughput 3D applications, further standardization for both assay development and data analysis have to be implemented.

The total number of cells needed, and the culturing method of organoids additionally hinder the performance of high-throughput screens, as it is difficult to work with ECMs in commonly used 384-well plates [222]. Furthermore, it has been shown that the artificial ECMs can negatively influence drug penetration by forming a protective barrier around the tumor cells, and thus lead to unreliable results during drug screening [230]. To circumvent these hurdles, drug screening protocols with tumoroid suspension cultures have been developed [219]. However, most studies present a far too small sample size for reliable translation of results into clinical practice [107].

The cells of the TME can also greatly influence the drug response of tumor cells [186]. However, as tumoroid cultures only maintain tumor cells of epithelial origin, the role of the TME cannot be modeled with conventional 3D organoid systems, highlighting the importance of using advanced co-culture and organ-on-a-chip models. However, establishing these advanced chip-based systems requires technical knowledge and specific instrumentation. Also, there are no standardized organoid-on-a-chip systems available yet, which makes validation of published data difficult.

Since the establishment and long-term cultivation of patient-derived organoids and PDTs has not been successful for some human tissues and cancer types, many researchers still rely on animal models. Besides using animal-derived tumoroids for cancer research, one of the most important model systems is the establishment of tumoroid xenografts to investigate the initiation and progression of human tumoroid-derived lesions in vivo. Although one of the limitations is the high number of tumoroids required for successful xenografting, established tumors stably recapitulate the morphology of the primary tumor. Additionally, this method leads to the development of cancer-specific symptoms in mice that can be seen less frequently in cell line-derived xenografts [248]. Animal models are a valuable tool for assessing the complex biological effects of drugs, which cannot yet be fully replaced by PDT models.

## Conclusion and outlook

As cancer is a highly heterogeneous disease, versatile models that can easily be manipulated in vitro, and stably reflect the characteristics of the primary tumor, are needed. Thus, tumoroids have a great potential to replace standard 2D cell culture or animal models. However, to adequately model the inter- and intra-patient heterogeneity, several tumoroid lines would have to be generated from the same tissue biopsy, which is not the standard practice [162, 163]. Additionally, limitations regarding tumoroid cultivation methods still need to be overcome. For example, artificial ECM hydrogels [257, 273, 274], or human-derived ECMs [275], have been developed to limit negative cross-species interactions for PDTs. Additionally, cultures with no matrix at all [276], or bioreactors [277] could be used for tumoroid expansion and drug screens in the future. Importantly, many groups have focused on the automation of 3D cell culturing and analysis methods for high-throughput applications to achieve a better translation of results to the clinic [278–280]. For high-throughput drug screens, protocols that either use tumoroid-derived single cells, or small- to medium-sized tumoroids in suspension, have already been developed [219]. Additionally, bioinformatic approaches and mathematical modeling have been combined with 3D cancer models to test for drug sensitivities faster [281–283]. To further improve the model systems available for developing and testing novel treatment options, multi-organ chip-based systems were developed, which are used to study the systemic effects of anti-cancer drugs on healthy tissue or other organs [284, 285], and thus could reduce the use of animal models for drug testing.

In the future, living biobanks of organoid and tumoroid models will be essential for real-time personalized treatment of cancer patients, as summarized in a recent review [286]. A number of clinical trials are already investigating the outcome of personalized therapy selection based on tumoroid drug sensitivity testing for several cancer types, including PaCa (NCT04931394, NCT04931381), BrCa (NCT04450706, NCT03544047, NCT05177432), lung cancer (NCT05136014), BlCa (NCT05024734), and several different cancers (NCT04279509) [287]. However, the standardization of methods has to be addressed by regulatory authorities and put into practice in the industry to successfully implement 3D models in the clinics. Open data platforms that include repositories of protocols, donors, organoids and tumoroids, and ethical information, are needed [288]. Such biobanks for human-derived 3D models have already been established by the National Cancer Institute in the US [289], and by a consortium of several European institutions (Human cancer models initiative) [290]. Recently, the Organoid Cell Atlas, which in addition to tumor samples and tumoroids also includes healthy organoids, was launched as a comprehensive database [291, 292]. Similarly, OrganoidDB has already collected bulk and single cell transcriptomic data of more than 16.000 organoid and tumoroid models for both human and mouse samples, in addition to data generated from primary tissue and cell lines for comparison [293].

Lastly, 3D cell models are a promising tool to reduce the number of animals used for preclinical cancer research in concordance with the 3R principles [294]. Due to a recent ground-breaking new law, the US Food and Drug Administration can now approve drugs for clinical trials on humans that were tested only in vitro on organ-on-a-chip models or organoid and tumoroid models [295]. Although alternative methods for drug testing, such as 3D cell models, are not yet standard practice, they hold great promise for redefining preclinical cancer research and precision medicine in the future, due to their molecular and phenotypic similarities to patient tumors.

## Data Availability

No datasets were generated or analysed during the current study.

## Abbreviations

ALI: Air-liquid interface
BlCa: Bladder cancer
BrCa: Breast cancer
CAF: Cancer-associated fibroblast
CRC: Colorectal cancer
CTC: Circulating tumor cell
CTG: Cell Titer Glo
ECM: Extracellular matrix
EMT: Epithelial to mesenchymal transition
GEMM: Genetically engineered mouse model
HNSCC: Head and neck squamous cell carcinoma
iPSC: Induced pluripotent stem cell
NF: Normal fibroblast
NK: Natural killer
PaCa: Pancreatic cancer
PCa: Prostate cancer
PDT: Patient-derived tumoroid
SCLC: Small cell lung cancer
TAM: Tumor-associated macrophage
TME: Tumor microenvironment
TOBisMC: Thiol-reactive Organoid Barcoding in situ Mass Cytometry
